# Did national implementation of interpersonal counseling improve adolescent depression treatment pathways in primary care? Protocol for a longitudinal cohort study

**DOI:** 10.1186/s12889-026-26466-6

**Published:** 2026-06-10

**Authors:** Outi Linnaranta, A. Myllyniemi, E. Salusjärvi, N. Seilo, J. Kaljadin, T.-R. Kukko, S. Tani, R. Kaltiala, T. Koskinen, M. Karukivi, J. Karvonen, S. Karri, J. Oksanen, E. Heinonen, O. Kiviruusu, S. Therman, P. Berg, J. Lahti, E. Rissanen, J. Lammintakanen, K. Ranta, R. Autio, M. Marttunen

**Affiliations:** 1 Finnish Institute for Health and Welfare, Mental Health Team, Helsinki, Finland; 2 Itla Children’s Foundation, Helsinki, Finland; 3 University of Helsinki, Helsinki, Finland; 4 Faculty of Medicine and Health Technology, Tampere University, Helsinki, Finland; 5 Faculty of Medicine and Health Technology, Department of Adolescent Psychiatry, Tampere University, Tampere University Hospital, Tampere, Finland; 6 University of Eastern Finland, Kuopio, Finland; 7 Department of Adolescent Psychiatry, University of Turku and Turku University Hospital, Turku, Finland; 8 Department of Adolescent Psychiatry, Oulu University Hospital, Oulu, Finland; 9 Department of Psychology, University of Oslo, Oslo, Norway; 10 Adolescent Psychiatry, University of Helsinki and Helsinki University Hospital, Helsinki, Finland; 11 Department of Psychology and Logopedics, University of Helsinki, Helsinki, Finland; 12 Folkhälsan Research Center, Helsinki, Finland; 13 Faculty of Social Sciences, Tampere University, Tampere, Finland

**Keywords:** Adolescent, Depression, Prevention, Treatment, Effectiveness, Economic evaluation, Equality, Screening, Health services, Implementation

## Abstract

**Background:**

Implementation of evidence-based interventions is one of the proposed responses to increased demand for treatment of adolescent depression. While the efficacy of interpersonal psychotherapy to treat depression of adolescents (IPT-A) is well established, the shorter adolescent interpersonal counseling (IPC-A) was implemented in primary care in Finland due to better feasibility. We present a protocol for a prospective evaluation of whether implementation of IPC-A has improved the naturalistic treatment flow and outcome of adolescents with sustained depression. We will evaluate the comparative effectiveness and cost-effectiveness of IPC-A and other treatment options, as compared to no treatment of sustained depression.

**Methods:**

We will collect a prospective cohort of grade 7 to 9 adolescents (13 to 17-year-olds) in selected Finnish schools using convenience sampling (*n* = 9000). We will describe the comparative effectiveness of naturalistic treatment, with an aim to compare three groups as defined at 6 months (with IPC-A, *n* = 100; with other psychosocial treatment, *n* = 200; or no treatment, *n* = 100). *The primary outcome measure* will be the proportion of adolescents who received specialized psychiatric services by 2 years after baseline in each group. *Secondary outcome measures* will include longitudinal changes in PHQ-9-A scores by 12 months, positive mental health, social inclusion, and quality of life. *Cost-effectiveness* will be evaluated using survey data at 12 months, and an *economic evaluation* using register data and information on service use 12 months before and up to 10 years after baseline. A universal evaluation of all adolescents, independent of mood, will provide prospective description of adolescents a) with *sustained depression* over the follow-up period (Patient Health Questionnaire 9 items, adolescent version, PHQ-9-A ≥ 10 in two measurements over 6 months), b) with a self-reported need and motivation for support, c) with therapeutic intervention, and d) benefits and harms of treatment. We will describe the *treatment received* and predictors of treatment and outcome based on reports from adolescents, caretakers, and therapists, as well as electronic patient records. Impact of training in IPC-A on competence and access to treatment will be evaluated.

**Discussion:**

The study will describe need for, pathways to, and content of mental health services for depressed adolescents over the two years following national implementation of IPC-A. The results can improve equal access to care, and inform decision makers about the best practices for treatment of depression, including utility of the implementation of IPC-A.

**Trial registration:**

ClinicalTrials.com NCT06390462 registered 2024-03-19.

**Supplementary Information:**

The online version contains supplementary material available at https://doi.org/10.1186/s12889-026-26466-6.

## Background

Interpersonal psychotherapy (IPT) is a structured intervention, originally developed for adults [1]. IPT for individuals with major depressive disorder has been shown to be highly effective in reducing depressive symptoms and anxiety, as well as improving overall functioning [2, 3]. The acceptance and feasibility of IPT through adaptations for a variety of patient groups has been good [1, 4].

IPT has been adapted for adolescents (IPT-A) for age sensitivity. Effect sizes for IPT in adolescents are higher than in adults (number needed to treat in adolescents 2.91 and in adults 4.85) [3]. Four systematic reviews on efficacy of IPT-A have been published [5–8]. They present results from in total 9 randomized controlled trials (RCTs) on individual IPT-A [9–16]. In addition to RCTs, the review with the best coverage of the original studies summarized results from 10 open trials and case studies [8]. The trials have shown large effects of IPT-A on depressive symptoms and medium-sized effects on functionality [2, 5–8]. IPT-A has consistently been superior to less structured interventions, and equal to cognitive behavioral therapy (CBT) [8].

In general, the acceptability of IPT-A has been good: in a review, the all-cause discontinuation rates were 19/284 (6.7%) for IPT-A, and 39/254 (15.4%) for control conditions [6]. However, 12 sessions of IPT-A were not considered to be feasible in primary care [17, 18]. Two adaptations of IPT-A have been developed for use in primary level services or school health and well-being services. Interpersonal Psychotherapy-Adolescent Skills Training (IPT-AST) is a school-based preventative approach to manage depressive symptoms in adolescents, and includes 2 individual and 8 group sessions [1]. A recent meta-analysis [19] showed remarkable improvement in patients’ depressive symptoms after IPT-AST interventions (weighted mean difference, WMD = − 5.05).

A shorter version of IPT-A, interpersonal counseling for adolescents (IPC-A), is composed of 3 to 8 individual sessions [1]. It was developed in an effort to provide a low threshold intervention in primary care and school health services [9]. Feasibility of brief IPT [9] or IPC-A [17, 20, 21] has been good in four reports: two smaller studies where professionals in primary health care provided IPC-A or brief IPT to school children [9, 17], another where youth workers provided IPC-A to young people [20], and one where graduate psychology students provided IPC-A to undergraduate students [21]. Despite consistently good feasibility, efficacy assessments of IPC-A in the treatment of depressive symptoms and major depressive disorder remain preliminary [17, 20]. An important limitation of prior research is the lack of suitable inclusion and outcome measures when IPC-A is used for prevention of depression.

While worries about mood are common among youth, not all youth seek or receive treatment. In the 2017 round of the School Health Survey (SHS) in Finland [22], one third (29%, *n* = 39 881) of young respondents reported having been worried about their mood during the past 12 months, more so girls (44%) than boys (14%). Of them, one third had not talked about problems with mood with anybody (38%, *n* = 15 191), and this proportion was higher among adolescents in upper high school (41%) and among boys (44% vs. 36% among girls). While half (50%) of adolescents who reported a worry about their mood had received help from a professional at school and others outside the school (35%), one in ten had not received any help despite a subjective need and reported effort to receive help. The 2023 SHS showed that depressive symptoms had not improved after the pandemic: one in five of adolescents reported depressive symptoms over the past two weeks [23].

The prevalence of unmet need for support for depression has increased over the years [24–26]. Gender, minority status, area of residence, and disabilities are factors that are associated with unequal access to treatment and treatment outcome [26–32]. What is less well understood is the impact of therapist training and competence on outcome and mechanisms of change in therapy [32].

As a response to the increasing demand for mental health services for adolescents, the Ministry of Social Affairs and Health in Finland planned a national implementation of an intervention suitable for prevention and treatment of depression in primary care [33]. In the first phase, IPC-A was successfully piloted in school health services [17]. Encouraged by the results in the pilot study, IPC-A was selected as an intervention for a national implementation [18]. Currently, after three years of training professionals in primary care in IPC-A, the numbers of trained professionals are approaching the estimated need of therapists for prevention and treatment of adolescent depression in primary care (*n* = 1700) [34].

Depression in young people is also associated with high societal costs. A recent meta-analysis showed that direct costs of social and health care services in depressed adolescents are even 2.8 [95% CI 1,7–4,6] times higher than of non-depressed adolescents in a 12-month follow-up [35]. The estimate included only costs of health care interventions (inpatient and outpatient treatments, emergency treatments, medication, and diagnostic tests). A Finnish estimate of the 12-month median direct costs of treatment of depression in lower secondary psychiatric care starting from the first outpatient visit in 2022 was 6 618 € per year [36], with a mean annual number of 23.6 outpatient visits per adolescent [36]. Based on a Finnish register study, having a diagnosis of major depressive disorder at age 16 to 23 increased costs of health care services even in young adulthood [37]. More precisely, for adolescents with no psychiatric diagnosis in adolescence the cost of health care services at 29 to 33 years was 5 600 € per year. For those with a diagnosed depression in adolescence the cost of health care services at 29 to 33 years was over 15 000€ per year, and cost of care for physical diagnoses comprised 29.1% of the total cost [37].

A recent systematic review found that the economic evaluations of depression prevention in children and adolescents have been controversial [38]. Most of the research concerns CBT. Short-term evaluations with 1-year follow-ups have shown that universal or targeted school-based CBT interventions have not been cost-effective compared to usual care [39, 40]. In contrast, a 2-year follow-up study found that the targeted CBT was effective but more costly compared to usual care [41]. In that study, the incremental cost-effectiveness ratio was 13 586 US$ per Quality-adjusted life year (QALY). A long-term modeling study [42] found that group-based CBT can be cost-effective compared to no treatment. Based on the long-term modellings in other interventions [43–45], universal or targeted psychological interventions can be more effective and more costly than providing no treatment. In those studies, the incremental cost-effectiveness ratio (ICER) has varied from 5 400 to 14 875 US$ per disability-adjusted life- year (DALY). Universal and targeted prevention can be effective but will also have higher costs than the comparator [35, 39–41, 43–45]. The only cost-effectiveness study of IPT was done for a group-based intervention in adult prisoners with a major depressive disorder [46]. Overall, the cost-effectiveness depends on the decision-makers’ willingness to pay for that effectiveness. It seems that the longer the follow-up, the better the results of an economic evaluation can be. Long-term economic evaluation of the Finnish IPC-A intervention is especially interesting since the intervention was chosen for a large-scale nationwide implementation.

## Goal and aims

*The overall goal* of this research project is to increase knowledge about clinical utility of implementation of evidence-based interventions in primary care. While the national implementation of IPC-A has been done over years 2020 to 2023, this is a protocol for a critical evaluation of the impact of implementation on the naturalistic pathways to care and treatment outcome over the two years after the implementation. To this end, we propose a protocol to collect a prospective cohort of grade 7 to 9 adolescents (age 13 to 17 years), using convenience sampling in selected Finnish schools.

### Aims

1. We will describe the need and motivation for help and naturalistic treatment flow of adolescents during 6 months in a population with no previous treatment of depression,
2. In a longitudinal follow-up, we will evaluate the outcome of adolescents with depressive symptoms, including the outcome of those with no treatment, and the comparative effectiveness and cost-effectiveness of IPC-A and other psychosocial treatment as usual (TAU),
3. We will evaluate predictors of the treatment flow and adolescent outcome at 12 months and 2 years after baseline.

### Research questions

1. Do young people reporting depressive symptoms have equal access to IPC-A or other psychosocial treatment in a 6-month follow-up, dependent on their risk for depression and protective factors, and independent of their area of residence, gender, or other minority status? (Aim 1)
2. Is IPC-A effective and cost-effective as compared to other psychosocial treatment in a 12-month follow-up? (Aim 2)
3. Can we identify adolescent-, family-, therapist-, or intervention-related factors which predict service utilization, outcome, benefits or harms of treatment after 12 months and 2 years? (Aim 3)

### Hypotheses

1. Availability of IPC-A improves access to treatment. This could be seen as a shorter time to treatment, and a larger proportion of or a broader diversity of adolescents with sustained depression being motivated to and/or receiving treatment (Aim 1, RQ1).
2. Adolescents who receive IPC-A have a better outcome, that is, are less likely to be referred to specialized psychiatric services and other social welfare and health services than adolescents with other psychosocial treatment or no treatment group (Aim 2, RQ 2).
3. Lower inclusion and positive mental health predict worse outcome, independent of depression, and higher adolescent inclusion, therapists’ sense of skillfulness and positive mental health, and training in IPC-A or other psychotherapeutic techniques are further predictors of better outcome (Aim 3, RQ3).

## Methods

### Study design

This is a prospective cohort study with convenience sampling, where treatment is not under the control of researchers but the sampling is based on a convenience sampling method. Implementation of IPC-A took place over years 2020 to 2023, and the sampling of adolescents will be done over the two years after implementation to evaluate spontaneous provision of IPC-A. *Inclusion criteria*: We will include all adolescents attending grades 7 to 9 (age 13 to 17 years) in selected lower secondary schools. *Exclusion criteria* include psychiatric care within the past 12 months or receiving a psychosocial intervention (psychotherapy, IPC-A, any other therapy or support for depression from the same professional for > 3 meetings) during the 12 months before baseline, an inability to reliably understand the Finnish, Swedish, English, Sami, or Finnish plain language versions of the survey, not having access to digital devices to respond to a follow-up survey, or an adolescent’s or caretaker’s report on need for exclusion, such as another medical condition – including a mental disorder – where current active treatment could be endangered by research. Exclusion will be made by the researcher based on the survey response from the adolescent and caretaker.

### Recruitment and eligibility

The recruitment of adolescents will be done from four university clinic areas (Northern, Eastern, Central and Western Finland) to provide a representative national sample based on interest of schools. Two to three municipalities from each university clinic area have consented to the study.

With the consent of the school, all grade 7 to 9 *adolescents* (age about 13 to 17 years) in the school will have a session about mental health literacy skills (how to talk with friends about mental health, how to know when help by an adult is needed). The information will be adjusted for their age. During that session, the goals of the study will also be described. A similar session will be held for *caretakers*; the session will be complemented by messaging all parents a link to a video recording and supplementary materials for mental wellbeing online, as well as providing information about the study.

Subsequently, a research assistant (RA) will help adolescents fill in a digital survey questionnaire. Upon agreement with the recruiting school, this can take place either after the information session or in the classroom, in a context of a group discussion at a general level about the dimensions of mental health and wellbeing, and options for help for mental health.

## Sample size

To estimate the number of adolescents to be screened, in the absence of available Finnish data, we used the information from the Netherlands Mental Health Survey and Incidence Study (NEMESIS) for incidence [47] and prevalence findings from NEMESIS [48]. Based on these findings, we roughly estimated that the incidence of depressive disorders would be 2% [18].

For the current study, a conservative estimate is to screen 6 000 to 9 000 young people. We estimate this, with a 15% prevalence and 2% incidence, approximately 50% of adolescents not having previous treatment despite prevalent depression at baseline to the cohort, and a 70% consent rate, to be sufficient to recruit a minimum of 100 consenting adolescents to the IPC-A group, 200 consenting adolescents to a group with other naturalistically available psychosocial treatments, and 100 to the depressed/no treatment-group. The size of any of these three groups might be considerably larger, but we will finish recruitment once the minimum size of all groups has been reached. Enriching the sample for IPC-A using recruitment by IPC-A-professionals will be done if the sufficient sample size for IPC-A cannot be reached with convenience sampling.

## Measures and data collection

Data will comprise surveys for adolescents, caretakers, and therapists, as well as register data and, where necessary, complementary information from medical files on the timing, duration, and content of psychosocial support and overall treatment of depression (Table 1). The respondents will receive an online link to respond at their convenience. We will not have in-person meetings after recruitment and informed consent.

**Table 1:** Study flow and timing of data collection

Data source	Timing				
	Intake	3 months	6 months	12 months	2, 5, and 10 years
Comprehensive student survey	All	All	All	If sustained depression over 6 first months (PHQ-9-A ≥ 10 twice)	-
Biweekly short student survey	x	x	x	-	-
Caretaker’s survey	x		x	-	-
Therapist’s survey			x	-	-
Intensity of treatment	Medical files, adolescent, caretaker and professional’s report				-
Register data					x

### A comprehensive adolescent survey

Adolescents will complete a survey on sociodemographic information and self-evaluation measures at baseline, at 3 and 6 months. Those who show *sustained depression* (Patient Health Questionnaire 9 items, adolescent version (PHQ-9-A) sum scores ≥ 10 twice during the first 6 months after baseline) will also complete the comprehensive survey at 12 months. The comprehensive adolescent survey is available as a shorter version in plain Finnish language, based on the decision of school personnel.

### Outcome measures in the adolescents’ and caretakers’ surveys

We list here the self-report questionnaires (Table 2) that will be presented to adolescents and caretakers at the various timepoints. The questionnaires will be presented in Finnish, plain Finnish, Swedish, English, or Sami, as suitable.

**Table 2:** A summary of the content of the adolescents’ and caretakers’ surveys

Name of the scale	Short	Length	Rating	Time frame	Describes
The Patient Health Questionnaire 9 items, adolescent version*^1^	PHQ-9-A	9 items	0 to 3	2 weeks	Depressive symptoms
The child-friendly EQ-5D version*^1^	EQ-5D-Y	5 items + VAS	5 items 0 to 2; The EQ VAS	The same day	Quality of life
Generalized Anxiety Disorder Scale-7	GAD-7	7 items	0 to 3	2 weeks	Anxiety
Young Person’s Clinical Outcomes in Routine Evaluation-Outcome Measure	YP-CORE	10 items	0 to 4	1 week	Response to therapy
Do you feel lonely?^1^		1 item	0 to 4	No time frame	Perceived loneliness
The short Warwick-Edinburgh Mental Wellbeing Scale	SWEMWBS	7 items	0 to 4	2 weeks	Positive mental health
Experiences of Social Inclusion Scale	ESIS	10 items	0 to 4	No time frame	Social inclusion
3 × 10D Survey*	3 × 10-D	10 items	0 to 10	No time frame	Functionality
Questions about worry as well as need for help*^1^		0 to 10 VAS scales	0 to 10 VAS scale	No time frame	Need and motivation for professional help
Motivation for Youth’s Treatment Scale	MYTS	8	1 to 5	No time frame	Motivation to psychotherapy
Other questions					
Questions from the Finnish School Health Promotion survey*			Yes/no, open field	Current 12 months	Sociodemographic information, minority status Alcohol and substance use, self-harming behavior*^1^
Use of health and social welfare services*^1^			Yes/no, open field	12 months	Information to classify as IPC-A, other psychosocial treatment, and no treatment groups
Content and overview of treatment*^1^		2 + 10	yes/no, open field; 10 statements true/false	During the intervention	Benefits and adverse effects due to treatment, safety of contact*

Scales marked with an asterisk (*) will be used in the caretaker’s survey as a proxy version. Scales marked with ^1^ will be included in the shorter plain language version

The *Patient Health Questionnaire 9 items* (PHQ-9) is a depression severity measure based on DSM criteria. The items are answered according to the frequency of symptoms (0 = not at all, 1 = some days, 2 = more than half of the days, 3 = almost every day). A higher score is indicative of greater depressive symptomatology. The PHQ-9 is a unidimensional measure with good internal consistency, also in adolescents [49–52]. In adults, the optimal sum score cut-off is ≥ 10 [53]. The PHQ-9 classifies respondents’ sum scores into depression severity as follows: 0–4 indicates no depressive symptoms, 5–9 mild depressive symptoms, 10–14 moderate depressive symptoms, 15–19 moderately-severe depressive symptoms, and 20–27 severe depressive symptoms [53]. One study of 442 youth with major depressive disorder (aged 13–17 years) proposed a cut-off of ≥ 11 [49]. We will use the PHQ-9-A version ≥ 10 according to DSM-5 criteria, including irritated mood for adolescents.

The *child-friendly EQ-5D version* (EQ-5D-Y) is based on the EQ-5D-3 L, which is a widely used standardized generic measure of health-related quality of life (HRQoL), originally designed for adults. EQ-5D-3 L has been used internationally in many settings, such as in clinical trials and population surveys. The EQ-5D-Y was introduced by the EuroQoL Group in 2009 as a more comprehensible instrument suitable for children and adolescents than the original [54]. The EQ-5D consists of five questions about mobility, looking after oneself, doing usual activities, having pain or discomfort, and feeling worried, sad, or unhappy. Each item has three response alternatives. A question about general health is evaluated on a visual analogue scale (VAS) [55].

The *Generalized Anxiety Disorder Scale-7* (GAD-7) is a seven-item self-rated instrument used to assess symptoms of generalized anxiety. Each item asks the individual to rate the severity of his or her symptoms over the past two weeks. Response options are 0 = not at all, 1 = several days, 2 = more than half the days, 3 = almost every day. GAD-7 scores can be categorized into mild, moderate, and severe anxiety with sum cut-offs of 5, 10, and 15 (score range 0–21). At a cut-off point of 10 or greater, sensitivity and specificity exceed 0.80 [56]. The adult version has shown adequate psychometric properties among adolescents in clinical [57] and the general population, with a reliability 0.9 of the sum score [58].

The *Young Person’s Clinical Outcomes in Routine Evaluation-Outcome Measure (YP-CORE*) is a ten-item measure designed for use in the 11–16-year age range. It is adapted for young people from *The CORE-Outcome Measure* for adults that has both 34- (CORE-OM) and 10-item versions (CORE-10) [59]. The CORE-OM and CORE-10 were developed as screening measures for psychological distress, including items for well-being, psychological symptoms, functioning, and risk. YP-CORE is well accepted, and the rate of missing values is low. Internal consistency (α = 0.83–0.92) and test-re-test reliability is good (*r* = .69), and the results of confirmatory factor analysis (CFA) have supported a one-factor model. The YP-CORE showed good concurrent validity against two widely used symptom-specific measures (*r* = .62–0.87). The measure was sensitive to change, showing a larger effect size (d = 0.55) than the Beck Depression Inventory (BDI-21) and Beck Anxiety Inventory (BAI) (d = 0.31-0.50). The Finnish translation of YP-CORE has good psychometric properties [60].

*Perceived loneliness* is a simple measure of loneliness based on self-assessment including only one question. This single question has been found to have good content validity in older adults [61].

The *Warwick-Edinburgh Mental Wellbeing Scale* (WEMWBS) with both its 14- and short 7- item versions measures positive mental health, that is, holistic aspects of wellbeing, namely eudemonic and hedonic wellbeing as well as psychological functioning [62]. This questionnaire allows for measuring mental wellbeing as a partially separable concept from mental illness, and has favorable psychometric properties, especially an approximate normal distribution in the general population. We will use the 7-item version (the Short WEMWBS; SWEMWBS) that has been validated in a large Finnish dataset [63], and validations in adolescents have proved successful [64–66]. Using the SWEMWBS, we will be able to calculate mental health costs and quality-adjusted life years (QALYs) [67].

*Experiences of Social Inclusion Scale (ESIS)* is a brief self-evaluation instrument to assess self-reported experiences of social inclusion. It consists of ten statements that map the respondent’s feelings of belonging, the significance of what they do, and the possibilities for action. One extreme of the continuum formed by the answers represents the experience of non-participation/social exclusion and the other the experience of inclusion. The participation indicator is calculated by scoring claims: a higher score means a stronger experience of inclusion. The sum score is scaled to a range of 0‒100. ESIS has been developed in the Finnish Institute for Health and Welfare (THL) Social Inclusion Coordination Project (Sokra). The theoretical framework is the capability approach (CA). In the validation study of the Finnish version of ESIS in adults, results indicated good internal reliability and consistency (α = 0.89), and factor analyses suggested a one-dimensional factor structure for the ten items of the ESIS [68]. Validation in younger adolescents is missing.

*Functionality (3 × 10D Survey).* The 3 × 10D survey is a quality-of-life mapping metric. The instrument comprises ten dimensions describing the life situation. The dimensions are evaluated in relation to the present moment, their importance, and the future. The young person evaluates areas of life for how important they feel the dimensions are and how satisfied they are with their current state [69]. The scale is awaiting international validation but publications from Finnish datasets are in preparation.

## Sociodemographic information

The comprehensive survey will include sociodemographic information collected at baseline after informed consent and will include information on age, self-identified gender, sex assigned at birth, first language, other minority status (language, sexual orientation, ethnic, other), area of residence, and household composition. The questions are the same or modified from the SHS. Register data will complement and verify responses.

## Biweekly short survey

Adolescents will respond to a short online survey biweekly over a 6-month follow-up, independent of their mood. This short survey will include PHQ-9-A and questions on their subjective need for help, as well as on any received treatment of depression.

### Motivation to treatment

*Need and motivation to treatment* will be asked: Are you worried about your mood (yes/no). Those worried about their mood will further be asked: How willing are you to have help from others to improve your mood? This is done with a VAS with a scale from 1 to 10. This is complemented by *the Motivation for Youth’s Treatment Scale (MYTS)* [70], an intrinsic treatment motivation scale for youth. The questionnaire includes 8 questions answered on a scale from 1 (strongly disagree) to 5 (strongly agree), where higher ratings indicate higher motivation. Based on the participants’ answers a total scale and two subscales: Problem Recognition (4 items) and Treatment readiness (4 items) are calculated. The internal consistency of all three scales in young people was found to be good (α = 0.84 – 0.89) and Confirmatory Factor Analysis supported the two-factor model [70]. These findings were also replicated in another study, although caution in using the sum score is warranted due to low correlation between the subscales [71]. We will send this survey to adolescents who show twice a PHQ-9-A ≥ 5.

## Caretaker’s survey

One caretaker, upon acceptance and choice of the adolescent, will be sent a link to consent to and twice fill in a survey using a secure link (within one month from baseline and at 6 months from baseline) (Table 2). The caretaker survey will include sociodemographic information. The scales in the caretaker survey include proxy versions of the PHQ-9, EQ-5D-Y, and 3 × 10D questionnaires, and questions about the adolescent’s mood, need for treatment, and willingness for treatment. We will also ask whether they or the adolescent have sought and received help for the adolescent, and if yes, what kind of support they received; or if not, what kind of support they think would be necessary. At 6 months, where applicable, we additionally ask about benefits and adverse effects of treatment.

## Benefits and adverse effects due to treatment

Benefits and adverse effects due to treatment will be estimated with open field questions for the adolescent (“*Do you feel that attending interpersonal counseling was useful for you?” (yes/no)*,* Do you feel that attending interpersonal counseling was harmful for you? (yes/no)*,* “If yes*,* please describe how”.* Open field responses will be analyzed with thematic analysis. Ten additional statements concerning experience about treatment were formulated by the team. This format was selected while valid and gold standard instruments for evaluation of adverse effects of therapy have been reported to be lacking [72]. The comprehensive survey at 3, 6 and 12 months contains items for potentially adverse effects (substance use and self-harm). To complement adolescent view, we will ask about benefits and adverse effects in the caretaker and professional’s survey with similar questions.

### Adolescent follow-up at 12 months

A comprehensive adolescent survey will be repeated at 12 months for those who show sustained depression; no further follow-up will be done for those who were not depressed over 6 months.

*A depressed adolescent in subjective need for treatment* is an adolescent who reports a need for help or treatment of depression, and depressed adolescents who *received treatment* are those who over 6 months report receiving treatment, for whom the caretaker report at 6 months indicates treatment of the adolescent for depression, or where register data shows contact to social or health care services. This information might be complemented from medical records, therapist reports, and/or phone calls to informants, if necessary.

*Adolescents with sustained depression* (self-reported PHQ-9-A ≥ 10 twice over 6 months, including intake) will be classified in three groups for further follow-up

1. Adolescents who received prevention or treatment of depression including IPC-A during 6 months of follow-up as reported by the adolescent or a therapist, or evident from the medical files (aiming at *n* = 100 recruited in order of identification). If IPC-A is started within six months after intake, the adolescent will be classified into this group (1)
2. Adolescents who received any other treatment for depression (aiming at *n* = 200).
3. Adolescents with sustained depression, with no treatment at baseline or during six months of follow-up (aiming at *n* = 100)

### The therapist survey

The information collected with therapist surveys at 6 months in case the adolescent reports a contact with a professional for help in depressive symptoms is summarized in Table 3. The surveys include content for all professionals to be responded once. Professionals with a training in IPC-A respond once a survey on implementation support and focus of IPC-A. Finally, all professionals respond a survey after each treatment period per adolescent, and IPC-A-therapists only describe some content specific for IPC-A intervention of the adolescent.

**Table 3:** A summary of the content of the therapist’s survey

Characteristic or name of the scale	Short	Length	Rating	Time frame	Describes
A single therapist survey for all therapists					
Sociodemographic status		3 questions	Multiple choice	No time frame	Gender, age, maternal language
Equality		6 questions	Multiple choice	No time frame	Place of birth, minority status, language skills, working with a therapy interpreter
Skills		9 questions	Multiple choice	Currently	Professional training, complementary IPC training, working experience
Professional skills and competence		12 items	Sufficient, some, insufficient, not relevant for my work	No time frame	Professional skills in evaluating and supporting subgroups of adolescents
Trainee Current Practice Report	TCPR	22 items	0 (disagree) to 5 (fully agree)	No time frame	Therapist’s subjective skillfullness and difficulties in therapy work
A single survey for IPC-A therapists					
Normalization Measure Development questionnaire	NoMAD	20 items	5-Point Likert Scale	No time frame	Implementation process and support
Provider REport of Sustainment Scale	PRESS	3 items	0 (disagree) to 4 (fully agree)	No time frame	Sustained use of IPC-A
All professionals respond per each study participant with treatment of depression (IPC-A or other psychosocial treatment)					
The flow of therapy		Collected per each session	Date, PHQ-9-A-score		”Dosing”of and response to therapy
Content of therapy sessions		17 statements	Yes/no		Content of intervention
Response to therapy					
Benefits and adverse effects of treatment		2 statements	Yes/no, open field	No time frame	Beneficial and harmful impact of therapy
Therapeutic relationship		10 opposite statements	Suitable statement chosen	During the intervention	Professional’s view on adolescent experience about interaction (corresponds to adolescent and caretaker surveys)
The short Warwick-Edinburgh Mental Wellbeing Scale	SWEMWBS	7 items	0 to 4	2 weeks	Professional’s positive mental health
Professionals will respond in case the adolescent received IPC-A					
Correspondence of the adolescent’s IPC-A intervention as compared to the IPC-A manual, professional’s evaluation		20 statements	0 (disagree) to 2 (fully agree)	After IPC-A intervention	Therapist’s reflection on content as compared to the IPC-A-manual
		6 opposite statements	Suitable statement chosen		

#### A single therapist survey for all therapists

This survey will be sent to the therapists providing the main psychosocial support, including therapists providing IPC-A and other therapists. The survey will include information on their sociodemographic details, professional training, and specifically, the amount and quality of the training they have had in IPC-A or other psychotherapy.

We will use *the Trainee Current Practice Report (TCPR)*, which is a comprehensive self-report questionnaire on competence [73]. TCPR was developed to assess how therapists develop during their training. The questionnaire features items gauging both subjective skillfulness (10 items) and difficulties (12 items) in therapy work. In the Finnish arm of the SPRISTAD study, the data of 267 psychotherapist trainees (representing various background professions, therapy training programs, and universities) has yielded good reliabilities (α = 0.89 and 0.84) for the two scales, respectively [73].

## A single survey for IPC-A therapists

*The NoMAD questionnaire* is a set of 20 survey items that assess the implementation process from the point of view of professionals in healthcare [74]. The NoMAD instrument has shown good internal consistency (α = 0.89 to 0.921) and face validity in earlier studies conducted in the Netherlands [74–76]. The validation of the Finnish translation remains to be done in this study.

*The PRESS* consists of 3 survey items, and is designed to measure success of implementation in the sustainment phase [77]. PRESS showed excellent internal consistency (α = 0.947) in Rasch analysis (Person Separation Index of 0.826) [77]. The validation of the Finnish translation remains to be done in this study.

Finally, concerning each IPC-intervention with an adolescent, the professional will outline the correspondence of the adolescent’s IPC-A intervention as compared to the IPC-A manual; the 20-statements evaluation form was comprised based on the Finnish IPC and IPC-A manuals [78].

## All professionals respond per each study participant with treatment of depression (IPC-A or other psychosocial treatment)

The therapist will report the timing of therapy sessions, content of the intervention and response, including benefits and adverse effects of the intervention. Finally, the professional will complete the 7-item SWEMWBS to assess their own positive mental health each time they respond to a survey concerning a participating adolescent.

### Description of the intensity of treatment

Description (dosing) of treatment will be based on the adolescent’s, caretaker’s and therapists’ reports. Where necessary, the information might be complemented from medical records, and/or phone calls to informants. This helps in defining the adolescents as three groups (those who received IPC-A, other psychosocial treatment or no treatment), describing the overall use of resources, and calculating intervention cost for cost-effectiveness analysis at 12 months.

PHQ-9-A during the IPC-A intervention. We do not control provision or timing of IPC-A intervention as part of the study. However, adolescents in the IPC-A group will, even if the treatment period continues after six months, complete weekly reports of PHQ-9-A as it is an integral part of treatment, and this information will additionally be collected for the purposes of the study. Thus, even if IPC-A would continue after 6 months after baseline, we will be able to evaluate the response over the IPC-A period. 

## Register data

Register data will be collected to define health and social care service use and costs. Register-based data will include 12 months prior study to evaluate pre-trends of service use and costs. A further prospective register-based data collection for economic analysis will be done at 2 years, 5 years, and 10 years after baseline.

We will use four Finnish nationwide registers provided by THL: 1) Care Register for Health Care, 2) Register of Primary Health Care visits, 3) Care Register for Social Welfare, and 4) Register of Child Welfare. In addition, we will use the Drug Prescription Register (Statistics on reimbursements for prescription medicines and rehabilitation) from the Social Insurance Institution of Finland (Kela). The data extracted will include use of emergency services and hospitalizations, which might be due to suicidality, somatic complications from self-harm, or for psychiatric indications. Registers also cover use of inpatient and outpatient services, use of primary care, reimbursements for medicine expenses, provision of psychotherapy or neurocognitive rehabilitation, committed suicides, use of child welfare services and use of social services. 

## Outcome measures comparing three groups of adolescents with sustained depression (IPC-A, other psychosocial treatment or no treatment)

### Primary outcome measure

- proportion of adolescents referred to specialized psychiatric services during 12 months after baseline.

### Secondary outcome measures

- proportion of adolescents with any need for psychosocial or psychiatric treatment or social services at 2, 5, and 10 years after baseline.
- longitudinal changes in PHQ-9-A score by 12 months in each of the three groups in the repeated adolescent surveys.
- EQ-5D
- GAD-7
- YP-CORE-OM
- loneliness
- SWEMWBS
- ESIS
- 3 × 10D Survey

Complications and harmful behavior during the intervention (surveys and register data).

- alcohol and substance use.
- self-harming behavior and suicidality.
- hospitalizations during a therapeutic intervention.
- drop-out from treatment.

All analyses will be *intention-to-treat*, though loss to follow-up will be minimal, due to the outcome measures being comprehensive national registers. Cost-effectiveness will be estimated at 12 months and economic evaluation will be completed at 2 years, 5 years and 10 years after baseline.

## Statistical analysis

### Statistical analysis of psychometric measures

For descriptive purposes, we will use both parametric and non-parametric descriptive statistics. Tests for non-parametric measures will be used for testing statistical significance of correlations and between-group differences (IPC-A, other psychosocial treatment, no treatment), with the alpha level set to 0.05 in each analysis separately. Conventional psychometric characteristics of all measures will be described and compared between self-identified cis-male and cis-female participants. If the size of a subsample is > 20, we will additionally evaluate adolescent groups by gender, sexual orientation, report of ethnic and other minority status, specific groups of first language, or plain language.

To evaluate the validity of each scale, we will analyze the proportion of missing responses. Since there is some indication about limited measurement precision among adolescents for some response options [79], we will use item response theory (IRT) or similar methods to take into account separate probabilities for response option of each item. In the psychometric analysis of the individual scales, we will use confirmatory factor analysis of categorical items to test for psychometric properties. We will also correlate PHQ-9-A with each of the other scales (GAD-7, YP-CORE, perceived loneliness, WEMWBS, EQ-5D-Y, ESIS, and 3 × 10D Survey). In the longitudinal survey dataset (baseline, bi-weekly measurements over 3 months, comprehensive surveys at baseline, 3, 6, and 12 months), we will estimate test-retest reliability and sensitivity to change of the scales by observing change in PHQ-9-A in relation to changes in other scales.

We will correlate adolescents’ responses with caretaker and professional responses and use caretaker and professional responses to verify and complement adolescent responses. The bi-annual SHS is a national survey, with the last round completed in spring 2023. Where possible, we have included the same questions as the SHS and will thus be able to estimate how representative our cohort is compared to mean values in the municipality.

#### Statistical analysis of primary and secondary outcomes

In the analysis for outcome and predictors of outcome, after observing the data, we will use propensity score matching to balance for systematic differences between treated and non-treated adolescents. We will compare adolescents in three groups (IPC-A, other psychosocial treatment, or no treatment group, as defined at 6 months). Follow-up with surveys will continue for up to 12 months and with register data for up to 10 years.

We will use PHQ-9-A at baseline and mixed linear modelling for individual outcome for PHQ-9-A during the 6 months of follow-up and test whether the three groups (IPC-A, other psychosocial treatment, and no treatment) differ in variation of symptom severity and outcome in follow-up.

## Predictors

We will further describe what adolescent characteristics are predictors of motivation for help (expressed in bi-weekly surveys or MYTS), need for help as expressed by the caretaker, of seeking treatment (bi-weekly surveys), or actually receiving treatment or psychiatric specialized care, or complications and harmful behavior during the intervention (hospitalization, suicide attempt, suicide, severe criminal behavior, or decision on the placement or taking into care by child welfare services). Predictors include sociodemographic factors, baseline symptoms (PHQ-0-A, GAD-7), positive mental health (SWEMBS), functionality and quality of life (YP-CORE, EQ-5D-Y, 3 × 10D Survey), as well as protective and risk factors (ESIS, perceived loneliness). We will also evaluate whether membership in some subgroups of > 20 adolescents (gender or sex, minority status, functional limitations, first language, area of residence) is predictor of subjective or parent-reported need for treatment, likelihood of actually receiving therapy, or type, content or success of treatment, or other factors above.

Other correlates of adolescent outcome include caretaker-reported family characteristics and correlations between adolescent and caretaker ratings for symptoms and functionality. Finally, we will evaluate whether therapist characteristics (sociodemographics, training, experience, specific skills, subjective competence (TCPR), and having an IPC-A therapist training or supervision, or implementation support (NoMAD, PRESS) are outcome predictors, independent from adolescent characteristics.

### Analysis of pathways to care

We will describe the proportion of adolescents who receive any support for depression and who receive IPC-A, as well as time to treatment or support (Kaplan-Meyer analysis).

We will characterize the schools and municipalities of recruitment based on the number of professionals overall, and therapists trained and providing IPC-A in relation to the number of adolescents at the school or municipality. This information will be used to evaluate the impact of availability of IPC-A therapists on likelihood of equally receiving any treatment for depressive symptoms, shorter time to treatment, and outcome of depressed adolescents recruited to the study (Hypothesis 1). Combining different evaluations, we will the estimate utility and added value of implementation of IPC-A.

### Economic evaluations

The overall goal of the economic evaluation of IPC-A is to estimate costs and cost-effectiveness of the IPC-A intervention to provide information for health and social care decision-makers. Our aim is to evaluate whether the IPC-A is cost-effective as compared to other psychosocial treatment and no treatment in a 6- and 12-month follow-up from a societal perspective. Also, we aim to evaluate the IPC-A intervention’s effect on health and societal care service use and costs of adolescents with depressive symptoms in 2-, 5-, and 10-year follow-ups compared to other psychosocial treatment and no treatment. The cost-effectiveness of the IPC-A intervention will be evaluated together with the effectiveness study at 12 months and therefore the study design will be as described previously. We will use the effectiveness data and registers of health and social care services and medical purchases collected as described above.

The cost-effectiveness evaluations will combine both effectiveness and costs information of the IPC-A as compared to other psychosocial treatment and no treatment. Two separate analyses will be conducted. First, we will use EQ-5D-Y-based QALYs as the effectiveness outcome. Second, we will use PHQ-9-A scores as the effectiveness outcome. We will apply the limited societal cost perspective including, for instance, intervention costs with relevant use of both health and social care services and medicine purchases from national registers. To enable the use of micro-costing, we will collect the resource uses from multiple sources and use National unit costs for health and social care services in monetarization [69]. Multiple imputations [80] will be performed for missing effectiveness outcomes and cost items if needed.

We will use standard methods of cost-effectiveness analyses, and will, for instance, apply incremental analysis of the effects and costs. First, we will assess dominance of the intervention, that is, whether an intervention is more effective and less costly. In case without clear dominance, we will calculate incremental net health benefit or net monetary benefit comparing the alternatives with 95% confidence intervals [81].

### Data management

### Data entry

Data will be collected using the THL in-house questionnaire engine Lomakepalvelu (questionnaire service).

### Data coding

All participants will receive automatically generated identifiers and additional, automatically generated identifiers for caretakers and professionals. All survey data will be stored using this research ID. Register data including person IDs will be managed using the Findata Kapseli service. Sensitive personal information will be stored separately from any questionnaire data. All other data will be stored pseudonymized.

### Data storage

Data will be stored on the THL database system OLAP server, where the survey data, sample data, and register data will be collected for analysis. Participant identifier data will be stored separately on the THL research and scheduling service “*Tutkimus ja ajanvarauspalvelu* TAP”.

### Data confidentiality

Subjects have no access to information provided from other linked persons. Data access is restricted by user roles with permission of PI. No access to participant level data is provided.

*Data monitoring committee* will be comprised of the senior researchers in the team (Linnaranta, Marttunen, Ranta, Lammintakanen, Heinonen, Lahti, Koskinen, Karvonen, Kaltiala, Oksanen) and will monitor the number of participants every three months (adolescents with subsequent depression who received IPC-A, other psychosocial treatment, or no treatment, caretakers and professionals) and missing data. They will make the final decision about finishing recruitment or changing procedures to enrich recruitment for any group to reach sufficient power to respond to the research questions. If necessary, these modifications will be reported to the ethics committee and in scientific reports. Additionally, the same committee will audit data collection procedures every 6 months. No independent auditing committee is nominated since the treatment is not under control of researchers.

### Dissemination policy

The local results will be communicated to school and healthcare professionals annually. The research team includes several important national and regional stakeholders, and the main results will be disseminated into clinical and national development of health services in real time. The public will be informed through media interviews and other forms of communication.

### Authorship eligibility guidelines

We will follow international and journal guidelines for authorship eligibility.

## Timeline

We have started recruitment of the prospective cohort in March 2024 and will finish 12-month prospective sampling and data collection by the June of 2026. We expect to collect the first register data at the end of 2026. We expect to report psychometric properties and intercorrelations of measures in the baseline survey data in 2026, 6-month follow-up data in 2027, and 12-month follow-up data in 2028. We expect to describe treatment flow by 12 months, treatment (IPC-A, other psychosocial treatment) effectiveness, and predictors of 12-month treatment outcome as well as cost-effectiveness in 2028. The economic analyses at 2-, 5- and 10-year follow-ups will be reported accordingly as the data will be ready to be collected from registers. All main results will be reported as peer-reviewed articles in suitable international journals.

## Discussion

This will be a large prospective and nationally representative cohort of up to 9000 adolescents. The overall goal of this project is to increase knowledge about the naturalistic provision, effectiveness, cost-effectiveness and predictors of successful provision of treatment for adolescent depression in primary care. This will inform further improvements in detecting adolescents who would benefit from early prevention of depression and improving equal access to treatment of depression.

In Finland, regular screenings for health of certain age groups are in use. Screenings are annually done by a public health nurse and in lower secondary school, additionally include a visit to a doctor at the 8th grade (at age 14–15).

The national implementation of IPC-A in Finland adds options for low-threshold support to an existing regular screening of adolescents for depressive symptoms. Overall, the national goal is an implementation of evidence based (EB) interventions in primary care to treat the most common symptoms, and the first aim was to train interventions to treat depression [18, 33]. In addition to the intervention training, professionals in social welfare and health care services were trained in detecting depressive symptoms. As a result, school health services will have increasing knowledge about how to manage mild to moderate depression [34]. One of the major goals of the national implementation was to ensure rapid access to treatment, and to reduce the pressure on specialized services [33].

Only a minority of trained IPC-A therapists in Finland are psychologists with a professional training in mental health services [34]. Most of therapists are public health nurses and school social workers, since they are the adults to first meet adolescents who potentially benefit from preventive interventions [34]. While adaptations of IPT-A (such as IPT-AST and IPC-A) provided by social workers or lay people have shown efficacy, a critical evaluation about the background professional training is necessary. Through training, the number of professionals capable of providing mental health services has increased and the professional profile broadened, while the challenge of prioritizing other tasks remains a major challenge to actually provide interventions [34].

Implementation of evidence based (EB) interventions is but one of the possible approaches taken to improve access to high quality treatment for depressed adolescents [82]. Improving care of depressed adolescents is an important goal as such but has been hoped to prevent more complex psychiatric illness and suicides. Some of the alternative approaches taken in previous studies to improve quality of care for adolescents in primary care have included, but not been limited to methods and training for screening of depression and suicidal ideation with psychometric screens and symptom measures [83–87], digital screening [84], phenotyping [88] or feedback [89], training professionals in use of depression treatment guidelines [90], organizing services according to a stepped care, staging or a stage-based stepped-care approach [91], using other approaches such as measurement-based care and quality improvement [92], quality integrated care [93], early intervention [76], integrated services [88], collaborative care programs [94], and improving acceptability through cultural adaptations of interventions [95, 96]. Shared decision making tools seem not to have been used with adolescents with depression [97] and participatory planning and evaluation of implementation projects are limited in number but have recently been suggested [98, 99].

Not all procedures have been efficient in improving access to or efficacy of treatment of depression, and some have been harmful [100, 101]. Universal prevention, that is, preventive interventions targeted to a certain age group without screening for risk of depression, have shown some efficacy but are also expensive [85, 102, 103]. The effectiveness of universal screening for depression in adolescents remains disputed [79, 97] and screening for suicidality does not add to accuracy after screening for depressive symptoms [78]. Furthermore, non-supported education of gatekeepers lacks effectiveness [84, 104, 105]. Similarly, no evidence was found for efficacy of community-based mental health interventions targeted for refugee adolescents [106] or of a universal mindfulness intervention [101]. Any intervention can be harmful, including harm due to resources used both of people implementing, providing and attending the intervention unless efficient [100, 101, 107]. This is why evaluation of effectiveness and cost-effectiveness is necessary for any large-scale implementation processes.

This will not be a randomized controlled study but a prospective convenience sample over the years following national implementation of IPC-A, and the treatment offered will not be under control of the research team. This methodological choice might decrease the power for estimating efficacy of IPC-A but will help to describe the real-world availability and effectiveness. Legally, all adolescents have equal right to receive treatment for mental health in Finland and the services are free of charge. A considerable lack of personnel is an important limiting factor in provision of treatment both in primary care and specialized services. We will have a heterogenous group of adolescents with treatments varying from one treatment session to intensive psychotherapy. We will use multiple sources of information to describe the content of treatment and other support for depressed adolescents. Part of adolescents are expected to receive medication, and some of them as the only treatment. Due to the national register for medication, this information can be collected reliably.

Inclusion of all consenting adolescents without previous treatment of depression, independent of need for treatment, will enable a sample representing naturalistic pathways to care and thus, correspond to real life, unlike traditional RCTs. This will also enable estimating the cost of no therapy for depressed adolescents. By grouping different components of support and treatment, we aim to describe which treatments and how commonly have been provided. The methodological choice of not controlling for provision of treatment was done on purpose to estimate real-life provision and effectiveness of IPC-A, to describe pathways to care, and to describe the treatment of those adolescents who do not receive IPC-A. This was considered as more useful to inform the future planning of national, largescale implementation projects than a strict RCT.

We will use symptom measures but will not conduct diagnostic interviews. Thus, we cannot test cut-off points as compared to a clinical diagnosis. This is chosen to enable a feasible large-scale follow-up of adolescents with digital surveys, in a wish to improve attrition rate and, accordingly, to evaluate feasibility of treatments and less of the research procedures. We will have access to any clinical diagnoses from registers and will be able to estimate receiver operating curves (ROCs) for subjective need for or actually receiving therapy.

Through the recruitment process and repeated evaluations, we will likely enrich the proportion of adolescents seeking treatment. We expect, however, that the limited resources will still provide a sufficient proportion of adolescents with sustained depression who do not receive treatment during the 6 months. Responding biweekly to the same questions on depression will be a challenge for adolescents. The register data will reliably complement information on the main outcome (received specialized care).

## Supplementary Information

Supplementary Material 1.

## Data Availability

No datasets were generated or analysed during the current study.
